# Machine learning: from radiomics to discovery and routine

**DOI:** 10.1007/s00117-018-0407-3

**Published:** 2018-06-19

**Authors:** G. Langs, S. Röhrich, J. Hofmanninger, F. Prayer, J. Pan, C. Herold, H. Prosch

**Affiliations:** Department of Biomedical Imaging and Image-Guided Therapy, Computational Imaging Research Lab, Medical University of Vienna, Währinger Gürtel 18–20, 1090 Vienna, Austria

**Keywords:** Decision support, Artificial intelligence, Computed tomography, Imaging, Informatics, Entscheidungsunterstützung, Künstliche Intelligenz, Computertomographie, Bildgebung, Informatik

## Abstract

Machine learning is rapidly gaining importance in radiology. It allows for the exploitation of patterns in imaging data and in patient records for a more accurate and precise quantification, diagnosis, and prognosis. Here, we outline the basics of machine learning relevant for radiology, and review the current state of the art, the limitations, and the challenges faced as these techniques become an important building block of precision medicine. Furthermore, we discuss the roles machine learning can play in clinical routine and research and predict how it might change the field of radiology.

## Zusammenfassung

Maschinelles Lernen gewinnt in der Radiologie rasch an Bedeutung. Es ermöglicht die Auswertung von Mustern in Daten aus bildgebenden Untersuchungen und Patientenakten für eine genauere und präzisere Quantifizierung, Diagnose und Prognose. Im vorliegenden Beitrag werden die für die Radiologie relevanten Grundlagen des maschinellen Lernens dargelegt und ein Überblick über den derzeitigen Stand der Wissenschaft, die Limitationen und die anstehenden Herausforderungen gegeben, da sich diese Technik zu einem wichtigen Baustein der Präzisionsmedizin entwickelt. Es wird erörtert, welche Rolle das maschinelle Lernen im Rahmen der klinischen Routine und in der Forschung spielen kann; darüber hinaus wird ein Ausblick darauf gegeben, wie diese Technik die Radiologie langfristig beeinflussen könnte.

Machine learning is a rapidly evolving research field attracting increasing attention in the medical imaging community. Machine learning in radiology aims at training computers to recognize patterns in medical images and to support diagnosis by linking these patterns to clinical parameters such as treatment or outcome. These methods enable the quantification of disease extent and the prediction of disease course with higher precision than is possible with the human eye.

## The emergence of machine learning in radiology

Two recent advances have further accelerated the development of machine learning in radiology. First, the acquisition volume of medical imaging data is accelerating. Worldwide, during 2000–2007, an estimated 3.6 billion radiologic, dental radiographic, and nuclear medicine examinations were performed per year [18]. Medical imaging data are expected to soon amount to 30% of worldwide data storage [9]. Second, recent algorithmic development in the machine learning field together with new hardware, such as powerful graphics processing units (GPU), have yielded a dramatic improvement in the capability of these techniques.

Here, we review the current state of the art and the possible roles machine learning can play in medical imaging covering clinical routine and research. After summarizing the basics of machine learning, we discuss the most pressing challenges and structure the review around four core questions:

1. How can machine learning serve as a tool to perform automated quantitative measurements in radiological routine?
2. Can machine learning contribute to research by expanding the vocabulary of patterns we can exploit for diagnosis and prognosis?
3. Can we effectively expand the evidence on which machine learning relies from controlled studies to large-scale routine imaging data?
4. What are the roles of machine learning in current radiology?

These questions are connected to a number of challenges, tackled by research at the interface of machine learning and radiology. They range from the availability of data and expert annotations, to the exploitation of partially unstructured data acquired during routine for learning, and the coping with noise in both data, as well as annotations.

## Machine learning

**Fig. 1:**
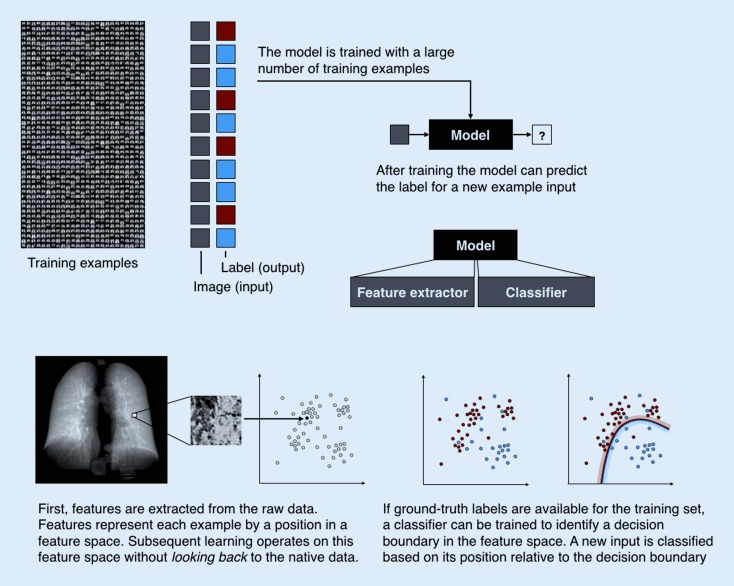
Principles of machine learning

In conventional analysis software, computers are programmed to carry out instructions specified by the developer. While machine learning consists of algorithms implemented in standard frameworks, its approach toward data analysis is different. Machine learning algorithms learn from examples and replicate the observed behavior—typically a mapping from an input to an output—on new data. The basic principles of such models are illustrated in Fig. 1.

For instance, training data can consist of example magnetic resonance imaging (MRI) volumes depicting segments of the breast. For each example, there is a label indicating whether there is a lesion or not in the volume. The algorithm learns a mapping from the input (volume) to the output (label).

This paradigm is called *supervised learning* since a large number of expert annotations in the form of correct labels are necessary. The resulting model is called a *classifier* since it classifies the input into a discrete set of possible classes (lesion, no lesion). After training, the classifier can process new input data and produce an output in the same categories as it has learned during the training phase. The first main component of these models is feature extraction or the mapping of raw input data such as lesion location or delineation to a feature representation, typically in the form of a vector. Features capture relevant characteristics of the raw input data. Constructing informative features for specific classification tasks was long the focus of research and has only recently shifted to being solved by algorithms. The second component is the mapping model, such as a classifier in the case of categorical output (benign vs. malign), or regression models (time to recurrence) in the case of continuous output.

By contrast, *unsupervised learning* does not rely on expert labels for the training examples. Instead, it processes a large number of unlabeled examples and seeks to find structure in the data. It can be in the form of clusters, groups of examples that are similar and clearly separable from other groups. This cluster model can then be used to assign cluster memberships to new data. Bone texture patterns that can be identified repeatedly across a population are such an example [22]. A different result of unsupervised learning is relationship patterns, often in the form of *manifolds* that reflect the fact that often different variables do not occur in arbitrary combinations; for instance, age, weight, and height in children.

### From feature construction to deep learning

A range of algorithms for classification and regression have been investigated over time, all of them sharing the aim of learning a mapping from a complex input space to a label or scalar variable. Nearest neighbor approaches predict labels based on the distance of the feature representation to those representations with a known label. Support vector machines [8] have proven immensely powerful in solving a variety of classification problems. They define the decision boundary in the feature space based on a small number of *support vectors*.

The insight that large heterogeneous training data are often beyond the capacity of single classifiers led to two approaches relying on *ensembles* of classifiers: *bagging* and* boosting*. Both use a large number of relatively simple models, so-called weak learners trained on parts of the data, and aggregate their estimates on new data, by mechanisms such as voting. During training, boosting builds a cascade of weak learners, each trained on data that previous learners were performing poorly on. An example is AdaBoost [7]. Bagging draws training examples and subsets of features randomly when training the ensemble of weak learners. A prominent example is random forests [3]. The latter introduced a reliable capability to estimate the information value of individual predictor variables for accurate classification. This led to a wide uptake in communities that mine large data for predictors, such as in genomics research, and to a shift in research efforts from hand-crafting features toward the exploration of large feature candidate pools and the algorithmic selection of predictive features by bagging.

The critical contribution of random forests was the ability of the algorithm to work with very large numbers of features, even if a portion of them is not informative, and to identify those that carry information during training. Algorithms that identify or construct relevant features based on examples were shown to outperform approaches that rely on the careful design of descriptors capturing relevant image information.

**Fig. 2:**
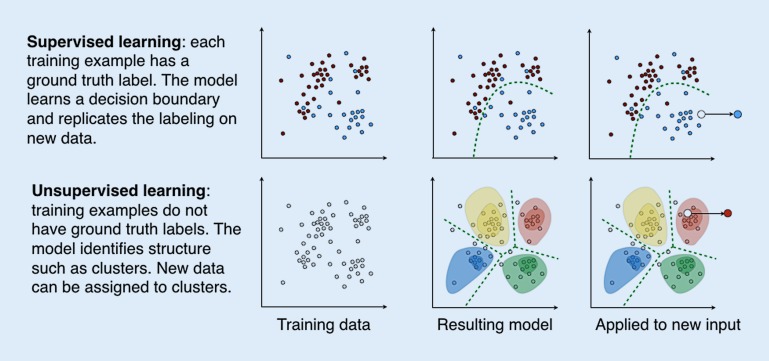
Supervised and unsupervised machine learning

**Fig. 3:**
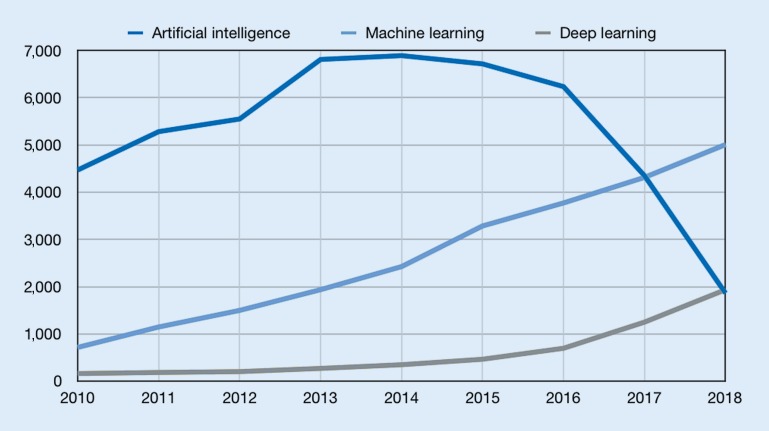
Number of publications for artificial intelligence, machine learning, and deep learning according to PubMed search. Values for 2018 are extrapolated numbers based on publications from January to April 2018

Most recently, deep learning has emerged as a powerful approach in machine learning. It uses neural networks with large numbers of layers to perform both feature construction and predictor learning simultaneously [16]. Convolutional neural networks were a first instance of such architectures used for supervised learning [15]. Unsupervised approaches include autoencoders (AE), a type of neural network that captures structure by learning to reproduce input such as images, through a bottle-neck low-dimensional representation [23], and more recently generative adversarial networks (GAN; [6]). With its expanding capabilities, machine learning is an increasingly important part of research at the interface of computer science and medicine (Figs. 2 and 3).

## Roles of machine learning in medical imaging

The computational analysis of imaging data using machine learning is about to fundamentally change our ability to understand disease, risk, and treatment. It will likely serve as the basis for the development and implementation of novel treatment strategies as well as the individualized, effective, and early delivery of care and preventive medicine. Linking research in machine learning and medicine will act as a powerful driver in this endeavor. Interdisciplinary collaboration is the key to creating better models to predict disease course and risk in individual patients, to forecast the response to a growing landscape of possible treatments, to deliver early prevention, and to make possible the optimal care for each individual. We will discuss two important roles of machine learning in radiology:

1. The automation of repetitive tasks, the enabling of radiomics, and the evaluation of complex patterns in imaging data not interpretable with the naked eye.
2. The discovery of new marker patterns and disease signatures in combined imaging and clinical record data, and the linking of these signatures to disease course and prediction of treatment response.

## Machine learning for computational quantification during routine

Machine learning can automate repetitive tasks, such as lesion detection, and the quantification of patterns such as textures that are hard to discern reliably with the human eye.

### Automation of repetitive tasks

One example of a tedious task is the detection, counting, and measurement of lesions in large-field-of-view imaging data before and after chemotherapy to evaluate response. Accuracy has a high impact on the prognosis and further treatment of the patient. Nevertheless, the task itself does not require a high level of experience but rather a high level of concentration. Additionally, finding a lesion and classifying it as responsive or not responsive to treatment is not sufficient, as a quantitative measurement may provide a more accurate description of the patient’s condition. There are already several software packages available for clinical implementation [20]. Using this software, the radiologist is able to supervise the results, include a complete quantitative summary in the radiology report, and focus on interpreting the findings in correlation with the clinical information. Typically, these approaches are based on supervised learning and rely on large manually annotated training corpora. Recently, approaches to exploit clinical routine imaging and record information to train such detectors have shown promising results. They rely on weakly supervised learning to link information in images and radiology reports [10].

### Using radiomics features to quantify subtle image characteristics

*Radiomics*, or the high-throughput extraction and analysis (or mining) of radiological imaging data [5], is a step beyond automating what can be done with the naked eye. In this process, hundreds of imaging features are calculated mathematically. They represent information that is not intuitively recognizable. Thereby, a large amount of quantitative information that was previously not accessible for human interpretation can be exploited. The resulting imaging features belong to specific groups (e. g., shape- and size-based or textural and wavelet features, as well as first-order statistics). While the choice of features to extract and the subsequent conventional statistical analysis may be done manually, a machine learning approach greatly improves the workflow by automatically extracting and selecting appropriate and stable features. Ultimately it can create predictive models that rely on features extracted from imaging data and corresponding clinical records. At this time, the main field of application of radiomics is oncology and the capture of fine-grained characteristics of lesions, leading to insights such as a correlation of radiomics features and the gene expression type of the imaged lung cancer [1]. However, this approach has the potential to improve the evaluation and computational assessment of other diseases too. Examples are chronic obstructive pulmonary disease [19] and osteoporosis [21].

## Machine learning as a tool for discovery

In addition to quantifying known patterns, machine learning enables the discovery of new patterns, which can serve as candidate markers for diagnosis, outcome prediction, or risk assessment.

### Evaluation of complex patterns and the discovery of new marker patterns

The diagnosis of disease patterns in radiology relies on the identification of imaging patterns that have proven to be of diagnostic and/or prognostic relevance. One prerequisite of such an approach is that the patterns must be defined precisely enough for trained radiologists to be able to recognize them with low interobserver variability. The reliable recognition of disease patterns in radiology is a challenging task and requires the systematic analysis of images. A good example of this is the diagnosis of the usual interstitial pneumonia (UIP) pattern, which is one of the most important patterns in diffuse parenchymal lung diseases. The diagnosis of a UIP pattern relies on the presence of reticular abnormalities and honeycombing in a basal and peripheral predominance [17]. In addition, there should not be any features more consistent with an alternative diagnosis. Although the definition of a computed tomography (CT) pattern of UIP appears to be straightforward, a number of publications showed that the interobserver variability of radiologists is moderate at best [24, 25]

Machine learning enables us to utilize imaging information that is not recognizable for the human eye and thus new disease patterns and predictive markers can be discovered. In lung imaging, machine learning is a promising approach to support the CT diagnosis of a UIP pattern and other patterns of diffuse parenchymal lung diseases and to predict the course of these diseases [2]. The potential was elegantly shown in idiopathic pulmonary fibrosis, where the increasing pulmonary vessel volume represents a stronger CT predictor of functional deterioration than traditional imaging patterns [13]. The strength of machine learning is that there is no constraint to described patterns. Instead, machine learning can identify patterns that are recognizable with high reliability, which could then serve as a basis for the diagnosis and prognosis.

Bone diseases are one example, where bone density is linked to outcome such as fracture risk but does not capture the rich variety of trabecular architecture linked to different diseases. Unsupervised learning is able to identify patterns that can be extracted repeatedly across individuals and even scanners [22]. Similar to image patterns, we can learn structure in the patient population. Here, at the patient level, unsupervised machine learning can identify clusters in chest CT imaging data linked to radiological findings, and even clinical parameters [11].

## The role of routine data in machine learning

The amount of available medical data has been growing exponentially over the past few decades, with approximately 118 million CT or MRI examinations per year in the EU [12]. We routinely acquire comprehensive information characterizing patients including results from multimodal imaging data; laboratory, physiological, and pathological results; and demographic data. Yet, we have only limited understanding of how to combine this complex information from multiple sources and link it to individual treatment response and patient outcome. Currently, only a small fraction of available data are used to collect evidence for supporting personalized medicine or to enhance clinical decision-making. The potential associated with these data is not yet fully reached.

*Clinical routine data* are a particularly valuable source of real-life patient histories and connected imaging data capturing disease course and treatment response across a wide variety of individual disease paths. Using routine data is challenging, since they are not acquired in a systematic fashion as during clinical trials, but instead with the sole purpose of individual patient care. Furthermore, parts of these data are unstructured, rendering their use for modeling challenging. Currently, only a fraction of routine imaging information is exploited for research and the development of models. While there has been a tremendous advance in image acquisition technology during the past five decades, the interpretation of imaging data has hardly changed. Radiologists focus their reports and summarize individual findings based on specific questions provided by referring clinicians (e. g., suspected pulmonary embolism). The information content of imaging modalities such as three-dimensional volumes with submillimeter slice thickness by far exceeds the capacities of visual assessment by humans. The complex multivariate relationship between clinical information (laboratory results, pathology results, clinical history) and imaging information is not sufficiently reflected in current studies. Large parts of multivariate information that is critical for personalized medicine are discarded, restricting evidence to small study populations rather than exploiting real clinical populations that are currently largely underexploited. There are two reasons for this: on the one hand the lack of technology to use heterogeneous and partly unstructured routine data for machine learning, and on the other hand the stumbling blocks of data management. Machine learning on this scale makes the integration of partially unstructured clinical information from patient records and imaging data necessary [4, 14], but to date technology for this is lacking. Both challenges are currently being tackled, and we can expect new kinds of evidence and robustness of prediction models once we are able to perform machine learning on this body of observations.

## The future

The increasing use of computer-assisted diagnosis software in radiology raises fear that such software might eventually take over the job of radiologists. There is no doubt that 5, 10, and 20 years from now, the job of radiologists will be very different. In a rapidly evolving specialty such as in radiology, this is to be expected. In fact, 20 years ago, the task of radiologists was different from what it is today and rapid technical developments are a hallmark of this field. Yet, the prime impact of machine learning will not be in replacing, but rather in enabling radiologists to use more powerful prediction models, and to rely on more comprehensive patterns that are informative for the individual disease course and treatment outcome. The radiologist’s role will be to develop such diagnostic paradigms, and to integrate these paradigms with overall patient care.

The primary role of machine learning will not be limited to the automation of specific analysis tasks, but will be critical in improving individual care by discovering new marker patterns and rendering these patterns useful. We will have powerful computational models that will enable a more accurate prediction of individual disease course, and which will forecast a response to treatment from observations such as imaging data and disease history. These models will facilitate the selection of personalized treatment strategies more effectively based on an assessment of each patient in light of an unprecedented scale of evidence from thousands of disease and treatment histories, together with models that translate these observations into an accurate prognosis.

As we become able to detect and quantify complex but subtle patterns in observations at an earlier stage, diagnostic categories might change. Through an understanding of the link between distributed signatures and prognosis, we will discover novel disease and response phenotypes, which, in turn, will contribute to research for novel treatments.

Until then, a number of hurdles must be overcome. Research communities that advance machine learning and medical disciplines such as radiology must collaborate more closely, learning to trigger algorithmic methodology by clinical questions and to ask new clinical questions whose answers will be made possible by computational algorithms. We will need to find methods to deal with heterogeneous multimodal data and the means for the efficient and possibly automated curation of such data. Algorithms will have to identify prognostic feature patterns in complex imaging data, and robust models will need to be able to predict, simulate, and assess the certainty of their output at the same time. While these challenges are significant, an increasingly joint effort by researchers across varying fields makes the solution to such challenges plausible, and the future of medical imaging might change more rapidly than we expect today.

## Practical conclusion

- Machine learning facilitates the exploitation of patterns in imaging data and patient records for more accurate and precise quantification, diagnosis, and prognosis.
- The computational analysis of imaging data using machine learning will change our ability to understand disease, risk, and treatment.
- Machine learning allows for the automation of repetitive tasks, the enabling of radiomics, and the evaluation of complex patterns in imaging data not interpretable with the naked eye. It leads to the discovery of new marker patterns and disease signatures in imaging and clinical record data, and the linking of these signatures to disease course and prediction of treatment response.
- Radiologists will be able to use more powerful prediction models and to rely on more comprehensive patterns providing information on the individual disease course and treatment outcome.

## Abbreviations

AI: Artificial intelligence. Machines that perform tasks typically requiring human intelligence, such as planning or problem solving
DL: Deep learning. An approach for performing ML, where deep neural network architectures are employed
ML: Machine learning. An approach for achieving AI, where instead of programming task execution by hand, the machine learns from examples
